# Biomechanical Analysis of Rectus Femoris Kinesio Taping Effects on Post-Muscle Fatigue Stop-Jump Task Performance

**DOI:** 10.3390/jcm13206277

**Published:** 2024-10-21

**Authors:** Wei-Hsun Tai, Chih-Yu Tu, Chih-Yen Tu, Chen-Fu Huang, Hsien-Te Peng

**Affiliations:** 1 School of Physical Education, Quanzhou Normal University, Quanzhou 362000, China; dlove520@hotmail.com; 2 Graduate Institute of Sport Coaching Science, Chinese Culture University, Taipei 11114, Taiwan; 3 Department of Physical Education, National Taiwan Normal University, Taipei 106209, Taiwan; 4 Department of Physical Education, Chinese Culture University, Taipei 11114, Taiwan

**Keywords:** quadriceps, lower limb, performance enhancement, athletic training, exercise physiology

## Abstract

**Objectives**: This study aims to compare the effects of kinesio tape (KT) on the rectus femoris muscle in athletes and novices under pre- and post-fatigue conditions. **Methods**: Nineteen male volunteers took part, and fatigue was assessed using the Borg CR10 Scale. Kinematic and kinetic data were collected using Vicon MX13+ infrared cameras (250 Hz) and Kistler force platforms (1500 Hz), respectively. Visual 3D v5.0 software analyzed the data, focusing on parameters like angular displacement, ground reaction forces (GRFs), impulse, and joint moments during a stop-jump task. A two-way mixed-design ANOVA was used to assess group, fatigue, and KT effects. **Results**: There was a significant effect after applying KT. The results showed significant differences in knee flexion range of motion (ROM), hip flexion moment, vertical impulse, and peak vertical GRFs between pre- and post-fatigue conditions (all *p* < 0.05). The trained group exhibited less knee valgus ROM, higher hip flexion velocity at initial contact, and prolonged time to peak proximal tibia anterior shear force. **Conclusions**: KT application was found to reduce lower limb loading, improve force acceptance and joint stability, and alleviate fatigue-induced disparities. These findings highlight the potential of KT in enhancing lower limb strength and performance, particularly under fatigue.

## 1. Introduction

Both muscle function decline and excessive external forces can severely impair athletic performance and lead to injuries, especially in activities involving high-intensity repetitive landing or jumping [1,2]. Notably, research indicates that nearly 70% of severe knee injuries in non-contact maneuvers are linked to anterior cruciate ligament (ACL) damage [3]. Fatigue exacerbates injury risks by reducing muscle strength and altering lower limb muscle activation patterns, which can disrupt joint kinematics [4,5]. This disruption increases ground reaction forces during landing and cutting maneuvers, heightening the susceptibility to non-contact injuries [6,7]. Recent studies emphasize neuromuscular fatigue as a critical factor in this process, with its adverse effects on muscle strength, joint stability, and coordination leading to an elevated risk of injury in the later stages of physical activity [5,6]. While ACL injuries are often highlighted, fatigue-induced muscle weakness can increase the risk of a range of injuries, further underscoring the importance of strategies to counteract fatigue or maintain joint stability during intense physical exertion [8,9].

In sports involving jumping, the landing phase of the stop-jump task presents a significant risk for lower limb injuries due to the demanding mechanics of rapid deceleration followed by a jump [10]. This combination of movements increases anterior tibial shear forces and can place excessive stress on the musculoskeletal system, particularly under fatigue conditions [6,11]. Research suggests that greater muscle strength and knee joint mobility contribute to reducing ground reaction forces during the stop-jump task [12]. However, fatigue-induced muscle weakness can disrupt lower limb joint mechanics, altering movement patterns and increasing injury risk [7,11,12]. Common biomechanical changes under fatigue include excessive dynamic knee valgus [6], poor trunk control [13], and altered joint kinematics such as excessive knee flexion [14], all of which heighten the risk of lower limb injuries. Therefore, strategies aimed at mitigating the effects of fatigue or enhancing joint stability during the stop-jump task are crucial for reducing injury risk in high-intensity sports.

Amidst similar physical exertion levels, ACL injury susceptibility rises with prolonged exercise. Athletes often use kinesio taping (KT) to reduce joint loading or delay fatigue onset. KT is widely used in sports medicine and rehabilitation to treat musculoskeletal issues [15]. It helps by expanding range of motion, reducing pain, and supporting joint and muscle function [16]. Numerous studies suggest that KT can bolster muscle activation in healthy athletes, augment muscle reaction speed, and refine proprioception [17,18]. For instance, research suggests that KT’s ability to provide proprioceptive feedback through skin stimulation aids in improving neuromuscular control and maintaining joint stability during physical exertion, especially under fatigue [19]. Nonetheless, the efficacy of KT in terms of enhancing muscle strength and joint mobility remains contentious [20,21,22]. Some studies have demonstrated its positive impact on muscle activation and performance, while others have reported minimal effects [23]. Furthermore, placebo effects may influence the perceived effectiveness of KT, as some athletes report performance improvements that may not be directly attributable to KT’s physical properties [24] and other factors such as experiment design, KT application techniques, or participant population demographics [25]. Nevertheless, some studies indicate that KT offers considerable benefits in cases of functional disorder or fatigue, particularly in reducing injury risk, while effectively stabilizing joints with minimal impact on the range of motion.

Previous research has primarily focused on the immediate impacts of KT, leaving gaps in understanding its sustained efficacy after fatigue. Definitive evidence for its effects across diverse demographic groups is lacking [26]. While KT shows promise in postoperative scenarios, its impact on lower limb muscle function in healthy individuals is unclear [22,25]. This study aims to compare the effects of KT on the rectus femoris muscle in athletes and novices under pre- and post-fatigue conditions. We hypothesize that KT will mitigate fatigue-induced biomechanical disruptions in lower limb kinetics and kinematics, including ground reaction forces and joint angles, during the landing phase of the stop-jump task.

## 2. Materials and Methods

### 2.1. Participants

Nine Division II-level basketball players (age: 21.7 ± 1.55; height: 176.95 ± 5.07; weight: 69.55 ± 5.85) who underwent regular training sessions of approximately 3 h per day for 5 days a week, and ten healthy students (age: 20.1 ± 0.83; height: 176.78 ± 3.58; weight: 72.33 ± 5.52) who engaged in regular exercise for approximately 1.5 h a day for 3 days a week were recruited and assigned to the trained and novice groups, respectively. The number of participants for each group was determined based on pilot data using G*Power software version 3.1.9.7 (ES = 0.61, α = 0.05, and β = 0.2) [27]. All participants had no history of orthopedic or neurological issues or lower limb injuries. Additionally, they reported no discomfort during the testing procedures. Written consent was obtained from each participant, and this study was conducted in accordance with the guidelines approved by the Human Ethics Committee of National Taiwan University (Approval number: 201412EM014).

### 2.2. Procedure

This research utilizes a randomized repeated-measures design to investigate the effects of KT on the rectus femoris muscle in athletes and novices under pre- and post-fatigue conditions. This study was structured in two separate sessions. In the first session, participants performed stop-jump evaluations without fatigue. In the second session, a lower limb fatiguing protocol was implemented to induce fatigue prior to the evaluations. Before data collection, participants underwent a warm-up that included a 5 min treadmill run at 6 km/h, followed by a 5 min dynamic stretching protocol targeting the major muscle groups of the lower extremities. Participants were also given time to familiarize themselves with the stop-jump task before formal testing commenced.

**Figure 1:**
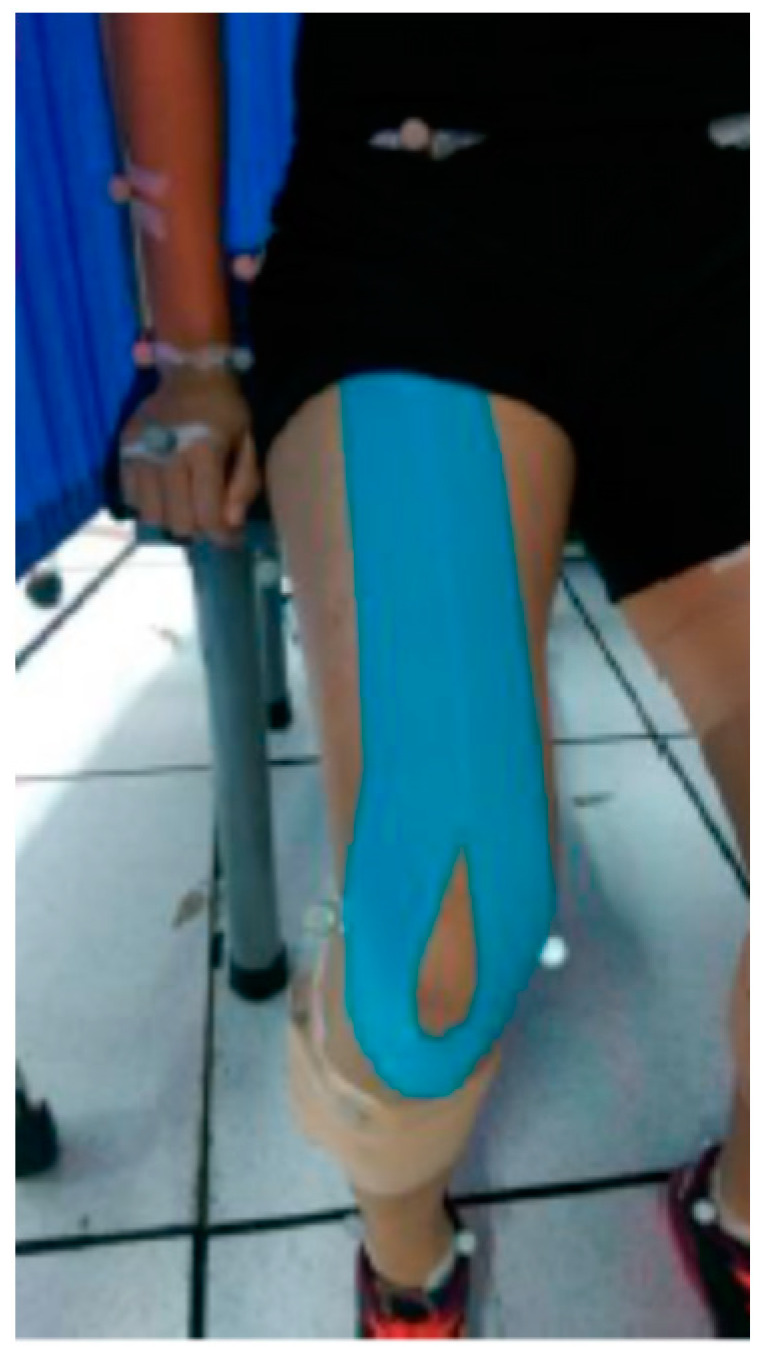
Application protocol of kinesio tape on the rectus femoris.

The fatigue protocol was followed in the second session. This protocol involved consecutive vertical jumps performed for 90 s (1 jump per second) until participants reached a state of volitional exhaustion, in accordance with the method described by Bosco et al. [28]. A set was terminated if a participant failed to maintain the metronome pace for five consecutive beeps or was unable to continue with the task. Verbal encouragement was provided throughout the protocol to ensure maximal effort. After completing the fatigue protocol, participants’ perceived exertion levels were assessed using the Borg CR10 Scale [29], requiring a rating of at least level 7 to be classified as fatigued. Following the fatigue assessment, certified athletic trainers applied kinesio tape to each subject, extending from the anterior inferior iliac spine to the tibial tuberosity, with a total tape length applied at 120% tension, using two I-shaped stickers covering the rectus femoris on both legs (Figure 1). Jump trials were then repeated five times for each condition, with a 1 min rest period between trials.

### 2.3. Data Collection

Kinematic data were captured using 10 infrared cameras operating at a frequency of 250 Hz (Vicon MX13+; Oxford Metrics Ltd., Oxford, UK), while kinetic data were gathered using two force platforms operating at a frequency of 1500 Hz (Kistler 9260AA6; Instruments, Inc., Winterthur, Switzerland). Fifty-three reflective markers were strategically positioned to track eight rigid segments representing the kinematics of the lower extremities. These markers were placed on specific anatomical landmarks for each segment. For the head, markers were positioned bilaterally on the temples above the skull and on the posterior aspect of the skull at the same horizontal level. For the trunk, markers were placed on the seventh cervical vertebra (C7), tenth thoracic vertebra (T10), clavicle, xiphoid process, and a reference landmark on the right posterior back. The upper limb markers were positioned on the acromioclavicular joint, medial and lateral epicondyles of the elbow, midpoints of the upper arm and forearm (tracking landmarks), medial and lateral aspects of the wrist joint, and on the proximal second metacarpal. In the pelvis, markers were placed on the anterior superior iliac spine (ASIS) and posterior superior iliac spine (PSIS). For the lower limb, markers were placed on the greater trochanter, midpoints of the lateral thigh and calf (tracking landmarks), medial and lateral condyles of the knee, and medial and lateral malleoli of the ankle. Finally, for the foot, markers were positioned on the medial head of the first metatarsal, proximal second metatarsal head, lateral head of the fifth metatarsal, and the heel. Subsequently, kinematic and kinetic data were processed using Visual 3D v5.0 software (C-motion, Rockville, MD, USA). The 3D trajectories of the markers and ground reaction force (GRF) data were filtered using a fourth-order zero-lag Butterworth digital filter with cut-off frequencies set at 10 Hz for marker trajectories [30] and 50 Hz for GRF data.

### 2.4. Data Analysis

Anatomical reference frames for the body segments were established with clarity: the positive x-axis (medial/lateral) directed towards the right, the positive y-axis (anterior/posterior) oriented forwards, and the positive z-axis (superior/inferior) directed upwards. The landing phase was defined as the period from the initial ground contact (IC) to when the attainment of maximum knee flexion angle was reached, while the take-off phase was spanned from the maximum knee flexion angle to the moment of take-off. Variables were analyzed for the leg that made initial ground contact (the first touchdown leg). Dempster’s normative anthropometric data were utilized to determine limb segment masses and moments of inertia [31]. Angular displacement, GRF, impulse, and joint moments were calculated to quantify biomechanical parameters indicative of ACL loading during the stop-jump task. Kinetic data were normalized to the participant’s body weight.

This study provided the participants’ demographics and various parameters using descriptive statistics (mean ± standard deviation). Statistical analysis was conducted using SPSS 18.0 software (SPSS, Inc., Chicago, IL, USA). A two-way mixed-design analysis of variance (ANOVA) was utilized, incorporating four within-subject factors of pre-fatigue, post-fatigue, and KT application on both pre- and post-fatigue conditions, alongside one between-subject factor of the group (comprising trained and novice subjects). This analysis aimed to assess the effects of KT, fatigue, group, and their interactions on all jump parameters. Bonferroni post hoc analysis was utilized to compare mean differences among conditions. Shapiro–Wilk test was utilized to assess the normality of data distribution. Statistical significance was considered for *p*-values less than 0.05. Effect sizes were determined using partial eta-squared (*η*^2^), with values of *η*^2^ = 0.01 indicating a small effect, *η*^2^ = 0.06 denoting a moderate effect, and *η*^2^ = 0.14 signifying a large effect [32].

## 3. Results

Table 1 presents the outcomes of interactions and group differences for key biomechanical variables. No significant interaction was detected between groups and intervention. However, significant main effects between interventions were observed for knee flexion range of motion (ROM), peak hip flexion moment, horizontal impulse in the first 50 ms after IC, vertical impulse in breaking phase, peak vertical GRF, breaking time, push-off time, total time, and jump height (all *p* < 0.05).

**Figure 2:**
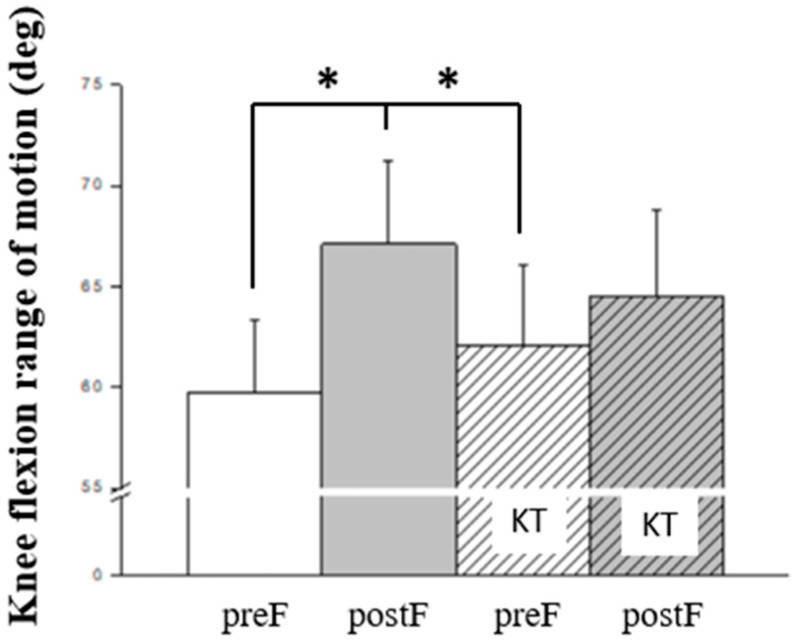
Bonferroni post hoc analysis of kinematics; KT, kinesio taping; preF, pre-fatigue; postF, post-fatigue; * *p* < 0.05.

**Figure 3:**
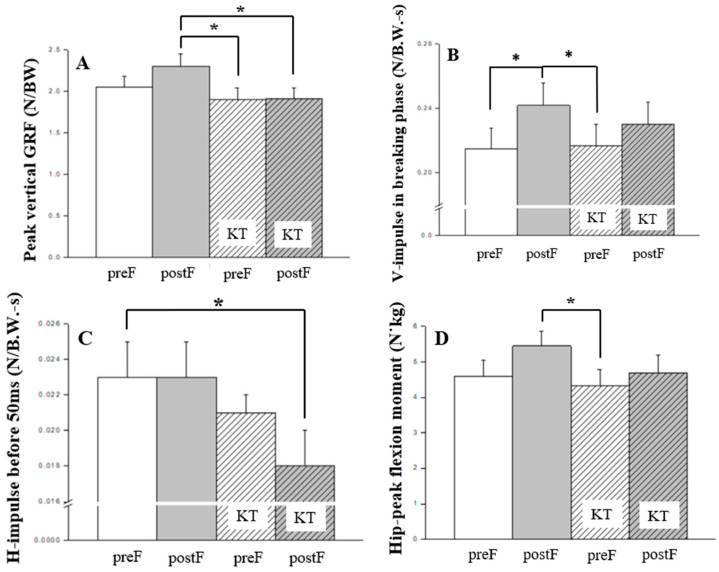
Bonferroni post hoc analysis of kinetics: (**A**) peak vertical GRF; (**B**) vertical impulse in breaking phase; (**C**) horizontal impulse before 50 ms; (**D**) hip peak flexion moment. GRF, ground reaction force; KT, kinesio taping; preF, pre-fatigue; postF, post-fatigue; * *p* < 0.05.

**Figure 4:**
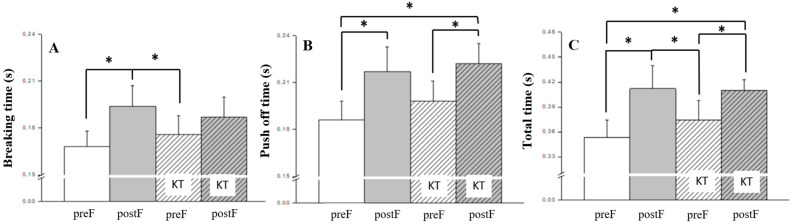
Bonferroni post hoc analysis of ground contact time: (**A**) breaking time, (**B**) push-off time, (**C**) total time. KT, kinesio taping; preF, pre-fatigue; postF, post-fatigue; * *p* < 0.05.

Bonferroni post hoc analysis was employed to further examine biomechanical variables related to kinematics (Figure 2), kinetics (Figure 3), and ground contact time (Figure 4). It revealed that knee flexion ROM, hip peak flexion moment, vertical impulse during the breaking phase, and peak vertical GRF were significantly greater in post-fatigue compared to in pre-fatigue conditions (all *p* < 0.05).

**Figure 5:**
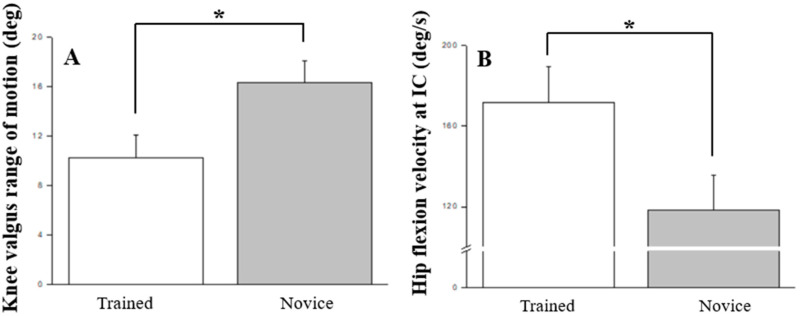
The kinematic variables that significant differences between novices and trained individuals: (**A**) knee valgus range of motion; (**B**) hip flexion velocity at IC. IC, initial ground contact; * *p* < 0.05.

**Figure 6:**
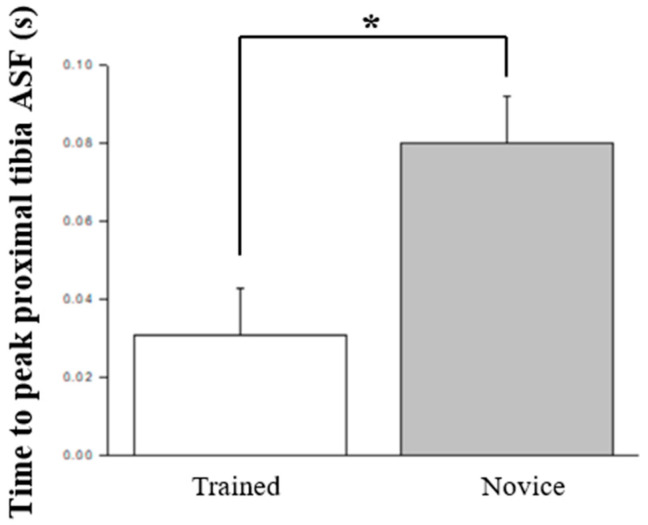
The kinetic variables that significant differences between novices and trained individuals; ASF, anterior shear force; * *p* < 0.05.

Table 2 presents the evaluations of pre-to-post changes with the application of KT intervention concerning major biomechanical variables in both trained and novice individuals (refer to Figure 5 and Figure 6). A statistically significant decrease in knee valgus ROM was observed within the trained group (*p* = 0.027, *η*^2^ = 0.257, power = 0.629). Additionally, compared to the novice group, the trained cohort demonstrated a significantly higher hip flexion velocity at IC (*p* = 0.050, *η*^2^ = 0.208, power = 0.513) and a prolonged time to reach peak proximal tibia anterior shear force (*p* = 0.010, *η*^2^ = 0.328, power = 0.774).

## 4. Discussion

The present study aimed to scrutinize the impact of KT on the stop-jump task subsequent to a fatigue-inducing intervention, comparing outcomes between professional athletes and typical college students. Findings unveiled that post-fatigue, the ‘stop-jump task’ prompted escalated lower limb loading and expanded joint range of motion, coupled with prolonged push-off duration. Nonetheless, application of kinesiology tape demonstrated a mitigated loading response, prolonged force absorption during ground contact, and augmented joint stability. While Csapo and Alegre [25] have underscored KT’s limited capacity to bolster muscle strength in healthy cohorts, our study illuminates its potential efficacy within the realm of human physical activity, particularly when fatigue serves as an intervention.

In the examination of kinematic variables, an increase in knee flexion range of motion was evident under the post-fatigue condition, aligning with prior research indicating fatigue-induced increases in knee joint range of motion [14,33], thereby elevating injury risk. Notably, when assessing KT application, no significant disparities emerged between pre- and post-fatigue conditions. This suggests that KT application indeed contributes to maintaining superior knee joint flexion performance, potentially reducing injury occurrence [21]. Furthermore, in the inter-group comparison during the stop-jump process, the training group displayed a smaller knee valgus range and a faster initial hip flexion velocity, indicative of enhanced knee joint stability. However, in the post-test following fatigue and KT intervention, no statistical differences were noted in knee and hip joint kinematic parameters. This implies that the use of KT may assist in mitigating the discrepancies in knee valgus angles due to varying skill levels or fatigue effects [19,34], thereby reinforcing KT’s stabilizing effect on knee joint dynamics among novice individuals and under fatigue conditions.

To explain the physiological mechanisms behind these findings, muscle fatigue can impair neuromuscular control and increase joint laxity, leading to reduced stability [6,35]. KT likely functions via both biomechanical and physiological means, providing proprioceptive feedback through skin stimulation, which enhances joint awareness and neuromuscular response under fatigue [21,36]. By stabilizing the joint, KT helps offset the effects of fatigue on motor control, preserving both kinematic and kinetic performance [37]. However, further research is needed to clarify how KT influences muscle spindle activity and central nervous system excitability in mitigating fatigue-related biomechanical changes. While KT shows promising outcomes, its effectiveness remains debated [38]. Variations in results may stem from differences in study populations, KT application sites, and fatigue interventions [23]. These conflicting findings highlight the need for the careful selection of participants and conditions to ensure KT’s application is tailored for optimal effectiveness.

Following the induction of fatigue, increased stress loading was observed across several parameters, including peak hip flexion moment, horizontal impulse within the first 50 ms of initial contact, vertical impulse during the braking phase, and peak vertical ground reaction force. With KT intervention, a significant reduction in lower limb stress, including impulse and peak ground reaction force, was observed. This highlights KT’s potential to alleviate post-fatigue lower limb loading. Such a reduction in stress may contribute to a decreased risk of ACL injuries, suggesting that KT could play a protective role in maintaining joint stability under fatigued conditions. While prior research has indicated the ineffectiveness of KT in enhancing muscle strength in healthy individuals or young athletes [20,25], suggesting that its tactile input may influence central nervous system excitability without significantly improving movement control [39], our findings present a contrasting perspective. The application of KT post-fatigue not only contributed to improved lower limb strength but also led to reduced loading across multiple stress parameters, particularly through its influence on both neuromuscular and biomechanical responses. This aligns with prior research that has reported similar benefits of KT application in populations with disabilities or chronic conditions [23].

In the stop-jump motion analysis of the present study, notable distinctions were observed in braking time, push-off time, total time, and jump height between fatigue conditions. Closer examination revealed that these primary differences stemmed from the fatigue intervention. Interestingly, the application of KT resulted in reduced braking time and increased push-off time, potentially enhancing jump performance, or possibly inducing a placebo effect from a psychological perspective. However, these effects did not fully manifest in the current study. In the overall execution of the stop-jump motion, both total time and jump height were significantly affected by fatigue, while KT use did not produce notable differences. This aligns with previous research, suggesting that KT’s impact on athletic performance may fall short of expectations [21,22,24]. Even if some effects are discernible, they may not significantly influence performance outcomes. For KT’s functionality, we strongly recommend its application in cases of functional impairment or excessive fatigue, primarily from a protective standpoint rather than as a way to enhance performance.

There are limitations that necessitate careful consideration in the present study. Firstly, it is pivotal to acknowledge that KT application was confined solely to the rectus femoris muscle, prompting concerns regarding the extrapolation of observed effects to other muscle groups. Second, the absence of specific performance tests for participant stratification into trained and novice groups introduces a level of variability that necessitates caution in drawing definitive conclusions. Third, this study primarily focuses on the acute effects of KT, offering immediate insights into its application and fatigue, but it raises concerns about the sustainability of these effects over time and their continuous efficacy during competitions, with there being a need for further longitudinal investigations. Fourth, while the incorporation of consecutive vertical jumps in the fatiguing protocol is relevant, it may not wholly replicate typical athletic scenarios, potentially compromising the ecological validity of this study.

## 5. Conclusions

This study investigated the effects of KT on stop-jump performance following fatigue in both trained and novice college students. After fatigue, lower limb loading and joint range of motion increased during the stop-jump task. The application of KT significantly reduced GRF loading, which may contribute to enhanced lower limb stability, particularly by maintaining knee joint flexion. Notably, the trained group demonstrated a smaller knee valgus range and faster hip flexion velocity during the stop-jump task, potentially indicating greater knee joint stability. Furthermore, kinematic analysis showed that post-fatigue KT intervention effectively preserved consistency in knee and hip joint movements compared to pre-fatigue performance, suggesting KT’s potential in mitigating the impact of fatigue. Despite certain limitations, these findings offer valuable insights into the positive role of KT in human physical activity, particularly under fatigued conditions.

## Figures and Tables

**Table 1:** Two-way mixed-design ANOVA analysis for key biomechanical variables in the stop-jump task.

	Major Biomechanical Variables	Main Effects		Interaction	
		*p*	*η* ^2^	*p*	*η* ^2^
Kinematics	Hip flexion angle at IC (deg)	0.111	0.123	0.266	0.075
	Knee flexion angle at IC (deg)	0.223	0.084	0.352	0.060
	Hip peak flexion angle (deg)	0.312	0.065	0.347	0.060
	Knee peak flexion angle (deg)	0.085	0.121	0.287	0.071
	Knee flexion range of motion (deg) *	0.015	0.184	0.735	0.024
	Knee valgus range of motion (deg)	0.395	0.056	0.959	0.006
	Knee internal rotation range of motion (deg)	0.419	0.049	0.438	0.047
	Hip flexion velocity at IC (deg/s)	0.394	0.056	0.979	0.004
	Knee flexion velocity at IC (deg/s)	0.649	0.031	0.696	0.028
	Hip peak flexion velocity (deg/s)	0.422	0.053	0.379	0.058
	Knee peak flexion velocity (deg/s)	0.921	0.009	0.744	0.042
Kinetics	Hip peak flexion moment (N/kg) *	0.017	0.179	0.179	0.091
	Knee peak flexion moment (N/kg)	0.219	0.087	0.831	0.007
	Peak knee anterior force (N/kg)	0.067	0.161	0.667	0.019
	Horizontal impulse in the first 50 ms after IC (N/BW/s) *	0.046	0.162	0.803	0.014
	Vertical impulse in the first 50 ms after IC (N/BW/s)	0.060	0.134	0.699	0.027
	Horizontal impulse in breaking phase (N/BW/s)	0.251	0.352	0.781	0.116
	Vertical impulse in breaking phase (N/BW/s) *	0.002	0.246	0.990	0.002
	Peak horizontal GRF (N/BW)	0.106	0.128	0.863	0.007
	Peak vertical GRF (N/BW) *	0.000	0.352	0.862	0.094
Performance	Time to peak proximal tibia anterior shear force (s)	0.133	0.111	0.203	0.089
	Time to peak knee extension moment (s)	0.838	0.415	0.399	0.049
	Breaking time (s) *	0.001	0.325	0.780	0.021
	Push-off time (s) *	0.002	0.294	0.217	0.085
	Total time (s) *	0.001	0.354	0.392	0.054
	Jump height (m) *	0.000	0.618	0.908	0.004

* *p* < 0.05. IC refers to initial ground contact, BW stands for body weight, and GRF represents ground reaction force.

**Table 2:** Pre-to-post changes in key biomechanical variables with kinesio tape intervention for trained and novice individuals.

	Major Biomechanical Variables	Trained	Novice	*F*	*p*	*η* ^2^	Power
Kinematics	Hip flexion angle at IC (deg)	61.75 ± 4.25	49.65 ± 4.03	4.279	0.054	0.201	0.497
	Knee flexion angle at IC (deg)	36.25 ± 3.16	30.69 ± 3.00	1.628	0.219	0.087	0.226
	Hip peak flexion angle (deg)	82.03 ± 5.81	69.90 ± 5.51	2.298	0.148	0.119	0.299
	Knee peak flexion angle (deg)	103.08 ± 7.72	93.10 ± 7.33	0.880	0.361	0.049	0.144
	Knee flexion range of motion (deg)	64.28 ± 5.42	62.44 ± 5.14	0.061	0.809	0.004	0.056
	Knee valgus range of motion (deg) *	10.24 ± 1.83	16.35 ± 1.73	5.888	0.027	0.257	0.629
	Knee internal rotation range of motion (deg)	19.06 ± 2.85	23.85 ± 2.71	1.488	0.239	0.080	0.210
	Hip flexion velocity at IC (deg/s) *	171.52 ± 18.23	118.47 ± 17.29	4.459	0.050	0.208	0.513
	Knee flexion velocity at IC (deg/s)	330.91 ± 34.98	273.23 ± 33.18	1.431	0.248	0.078	0.204
	Hip peak flexion velocity (deg/s)	330.29 ± 37.26	350.77 ± 35.35	0.159	0.695	0.009	0.066
	Knee peak flexion velocity (deg/s)	800.68 ± 67.92	685.13 ± 64.43	1.524	0.233	0.082	0.214
Kinetics	Hip peak flexion moment (N/kg)	5.03 ± 0.58	4.50 ± 0.55	0.440	0.516	0.025	0.096
	Knee peak flexion moment (N/kg)	2.79 ± 0.34	3.24 ± 3.23	0.919	0.351	0.051	0.148
	Peak knee anterior force (N/kg)	12.40 ± 1.38	11.98 ± 1.31	0.050	0.826	0.003	0.055
	Horizontal impulse before 50 ms (N/BW/s)	0.021 ± 0.002	0.021 ± 0.002	0.075	0.788	0.004	0.058
	Vertical impulse before 50 ms (N/BW/s)	0.058 ± 0.01	0.055 ± 0.01	0.182	0.672	0.011	0.069
	Horizontal impulse in breaking phase (N/BW/s	0.09 ± 0.01	0.08 ± 0.01	0.159	0.695	0.009	0.066
	Vertical impulse in breaking phase (N/BW/s)	0.23 ± 0.02	0.22 ± 0.02	0.416	0.527	0.024	0.094
	Peak horizontal GRF (N/BW)	0.877 ± 0.082	0.809 ± 0.878	0.364	0.554	0.021	0.088
	Peak vertical GRF (N/BW)	2.077 ± 0.189	2.004 ± 0.179	0.080	0.781	0.005	0.058
Performance	Time to peak proximal tibia anterior shear force (s) *	0.031 ± 0.012	0.080 ± 0.012	8.286	0.010	0.328	0.774
	Time to peak knee extension moment (s)	0.064 ± 0.009	0.075 ± 0.008	0.768	0.393	0.043	0.131
	Breaking time (s)	0.176 ± 0.016	0.187 ± 0.016	0.198	0.662	0.011	0.070
	Push-off time (s)	0.203 ± 0.20	0.208 ± 0.19	0.030	0.865	0.002	0.053
	Total time (s)	0.379 ± 0.035	0.395 ± 0.034	0.106	0.749	0.006	0.061
	Jump height (m)	0.468 ± 0.038	0.395 ± 0.036	1.891	0.187	0.100	0.255

* *p* < 0.05. IC refers to initial ground contact, BW stands for body weight, and GRF represents ground reaction force.

## Data Availability

All data generated or analyzed during this study are included in this published article.
